# Rankings Half-Yearly Abstract

**Published:** 1852-09

**Authors:** 


*Ranking's Half-Yearly Abstract.*

We have received from Lindsay & Blackiston No. 15, January to June, 1852, of this valuable work, we presume it is in the hands of most of our readers before this time. There is, perhaps no publication, either American or foreign, better calculated to keep the medical man acquainted with the improvements in our science; and few, if any, more widely circulated. For sale by Keen & Brothers, and booksellers generally. J.

